# ﻿New taxa and nomenclatural changes in *Bredia* (Sonerileae, Melastomataceae)

**DOI:** 10.3897/phytokeys.266.160564

**Published:** 2025-11-06

**Authors:** Jin-Hong Dai, Zhe Zhong, Ren-Chao Zhou, Ying Liu

**Affiliations:** 1 School of Life Sciences, Sun Yat-sen University, Guangzhou 510275, China; 2 State Key Laboratory of Biocontrol and Guangdong Key Laboratory of Plant Resources, Sun Yat-sen University, No. 135, Xin-Gang-Xi Road, Guangzhou 510275, China; 3 Guangzhou Baiyun Mountain Yuntai Scenic Area Management Center, No. 5, Yun-Shan-Nan Road, Guangzhou, China; 4 School of Ecology, Sun Yat-sen University, Shenzhen 518107, China

**Keywords:** *
Bredia
*, Melastomataceae, revision, taxonomy

## Abstract

A recent phylogenomic analysis has unveiled the first highly resolved phylogeny for the genus *Bredia*, clarifying long-standing uncertainties in species boundaries among taxonomically debated groups. Based on these findings, we propose a taxonomic revision encompassing the following updates: (1) *B. latisepala*, *B. longearistata*, and *B. longiradiosa* are synonymized under *B. esquirolii*; (2) *B. dulanica* C.L.Yeh, S.W.Chung & T.C.Hsu is incorporated within *B. hirsuta*; (3) two new combinations are established, alongside the formal recognition of three new species and one new variety (Suppl. material 1); (4) *Bredia bullata* S.Jin Zeng & N.H.Xia is regarded as a later homonym and therefore illegitimate name for *B. bullata* J.H.Dai & Ying Liu. Morphological descriptions, color plates, and updated distribution maps are provided for relevant taxa, supplemented by a key to all recognized *Bredia* species.

## ﻿Introduction

*Bredia* Blume (Sonerileae, Melastomataceae) was initially described based on *B. hirsuta* Blume, a species endemic to Taiwan and the Ryukyu Islands (Blume 1849). The delineation of generic limits within Sonerileae has long been complicated by significant homoplasy in morphological traits (Zhou et al. 2022), as reflected in the taxonomic history of *Bredia* (Blume 1849; Ito and Matsumura 1899; Li 1944; Chen 1984a, 1984b; Hansen 1992; Chen and Renner 2007). Recent molecular phylogenetic studies, however, have revised *Bredia*’s generic limit, excluding *Tashiroea* Matsum. ex T.Itô & Matsum. and incorporating additional species that were previously treated under *Phyllagathis* Blume (Zhou et al. 2019a, 2019b, 2019c). Today, *Bredia* is recognized as encompassing 27 species, distributed across central and southern mainland China, Taiwan, and northern Vietnam, with one species extending to the Ryukyu Islands in Japan (Zhou et al. 2018; Wen et al. 2019; Dai et al. 2020; He et al. 2020; Dai et al. 2022). While most species in the genus can be easily distinguished based on their morphological characteristics, defining species boundaries remains challenging for certain groups, including *B. esquirolii* (H.Lév.) Lauener, B. esquirolii var. cordata (H.L.Li) C.Chen, *B. fordii* (Hance) Diels, B. fordii var. micrantha (C.Chen) R.Zhou & Ying Liu, *B. longearistata* (C.Chen) R.Zhou & Ying Liu, *B. latisepala* (C.Chen) R.Zhou & Ying Liu, *B. tuberculata* (Guillaumin) Diels, and *B. yunnanensis* (H.Lév.) Diels.

**Figure 1.**
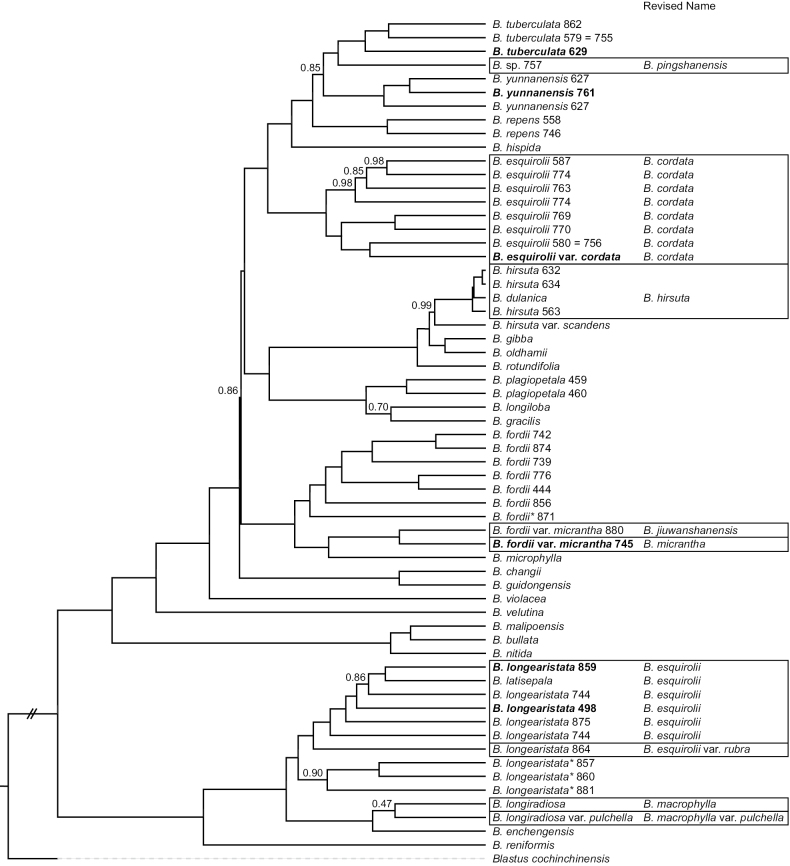
Coalescence species tree of *Bredia* inferred from 1,798 single-copy orthologs (SCOs), modified from Dai et al. (2025), showing the relationships among the target species. Local posterior probabilities are indicated above the branches for nodes without full support. Accessions collected from the type locality are noted in bold, those with possible hybrid origin are indicated by asterisks. Squares indicated the change of names upon revision.

The most comprehensive phylogenomic analysis of *Bredia* (Dai et al. 2025) resolved species delimitation through multi-population sampling of the taxonomically debated groups (Suppl. material 1). The integration of robust phylogenetic relationships (Fig. 1) and molecular/morphological divergence reveals: (1) *B. longearistata* and *B. latisepala* are conspecific with the types of *B. esquirolii* and *B. longiradiosa* C.Chen ex Govaerts, and the earliest legitimate name, *B. esquirolii*, should be retained for this unified taxon; (2) a population from Libo (LY 864) warrants recognition as a new variety within *B. esquirolii*; (3) B. esquirolii var. cordata (= *B. cordata* H.L.Li) and the populations (from Sichuan, Chongqing, and northern Guizhou) previously assigned to *B. esquirolii* constitute a species distinct from the type of *B. esquirolii*; (4) populations (from southwestern to southern Guangxi and southeastern Yunnan) formerly defined as *B. longiradiosa* differ from the type of *B. longiradiosa* and should be classified as a separate species; (5) two populations of B. fordii var. micrantha—one from the type locality of this name (LY 745) and the other from Rongshui County, Guangxi (LY 880)—are distinct from each other as well as from *B. fordii*, warranting recognition as separate species; (6) a population from Pingshan County, Sichuan (LY 757) represents a distinct new species closely related to *B. tuberculata* and *B. yunnanensis*.

**Figure 2.**
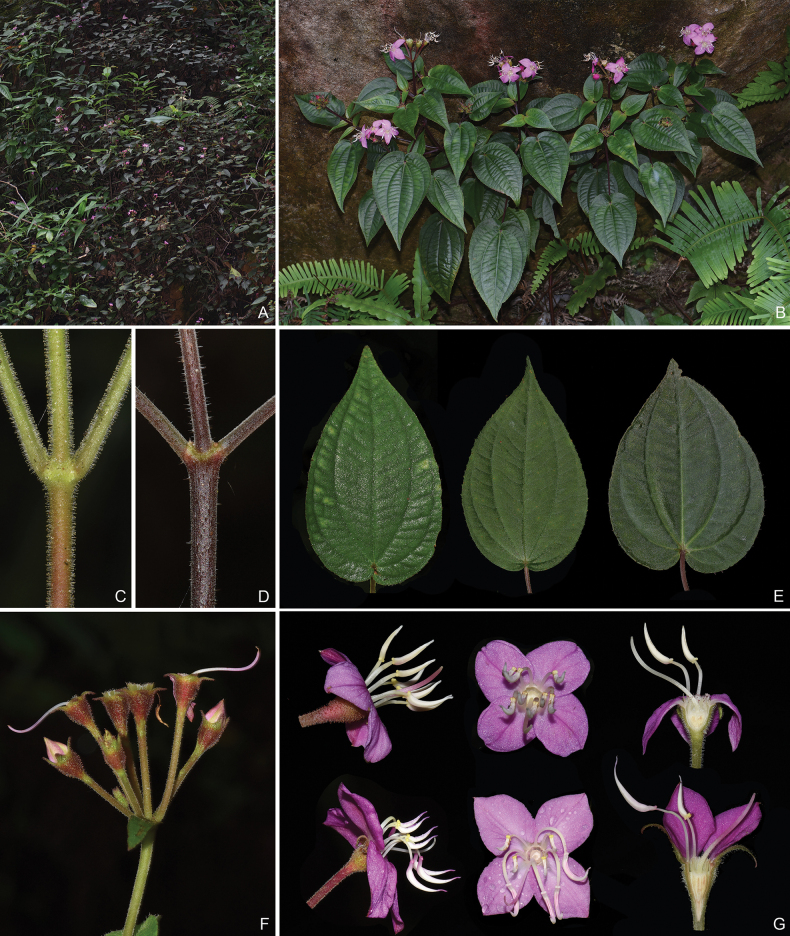
*Bredia cordata*. A. Habitat; B. Habit; C, D. Indumentum on the branchlets; E. Leaf shape; F. An inflorescence; G. Intraspecific variation of stamen morphology.

**Figure 3.**
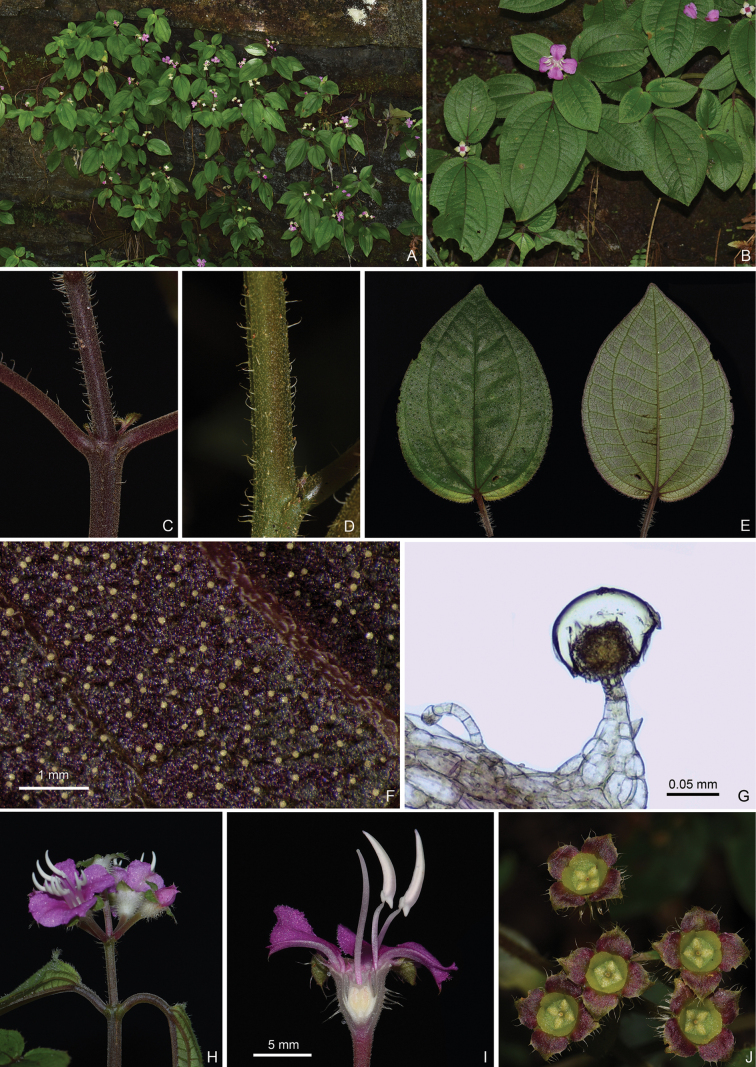
Bredia esquirolii var. esquirolii. A. Habitat; B. Habit; C, D. Indumentum on the branchlets; E. Leaf morphology; F. Yellowish glandular hairs on the leaf abaxial surface; G. Microscopic characters of the glandular hair; H. An inflorescence; I. Longitudinal section of a flower; J. Top view of the young capsules. Scale bars: 1 mm (F); 0.05 mm (G); 5 mm (I).

**Figure 4.**
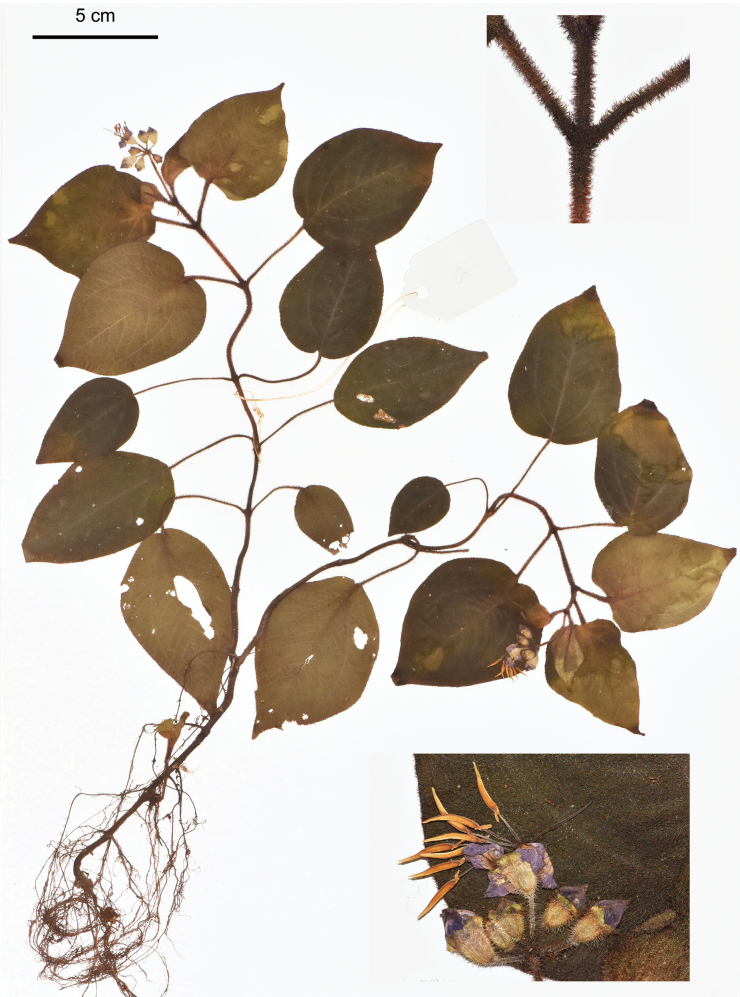
Holotype of Bredia esquirolii var. rubra, J.H.Dai and Y.Liu 864 (PE). The insets show details of indumentum and flowers. Scale bar: 5 cm.

**Figure 5.**
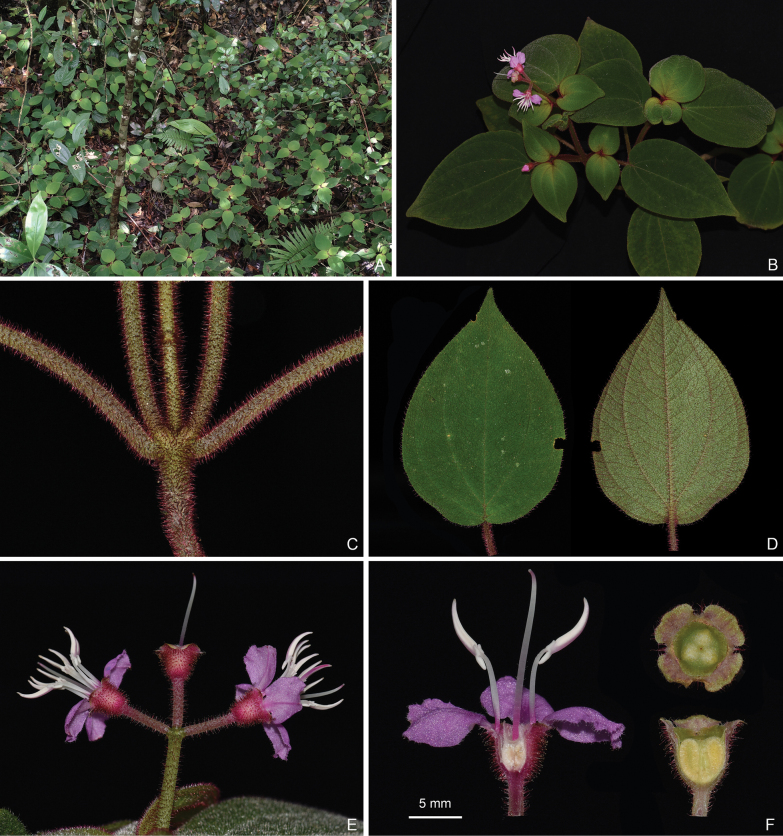
Bredia esquirolii var. rubra, all from J.H.Dai and Y.Liu 864 (A, PE, SYS). A. Habitat; B. A flowering branch; C. Indumentum on a branchlet; D. Leaf morphology; E. An inflorescence; F. Longitudinal section of a flower (left), top view (upper right) and longitudinal section (lower right) of young capsules. Scale bar: 5 mm (F).

**Figure 6.**
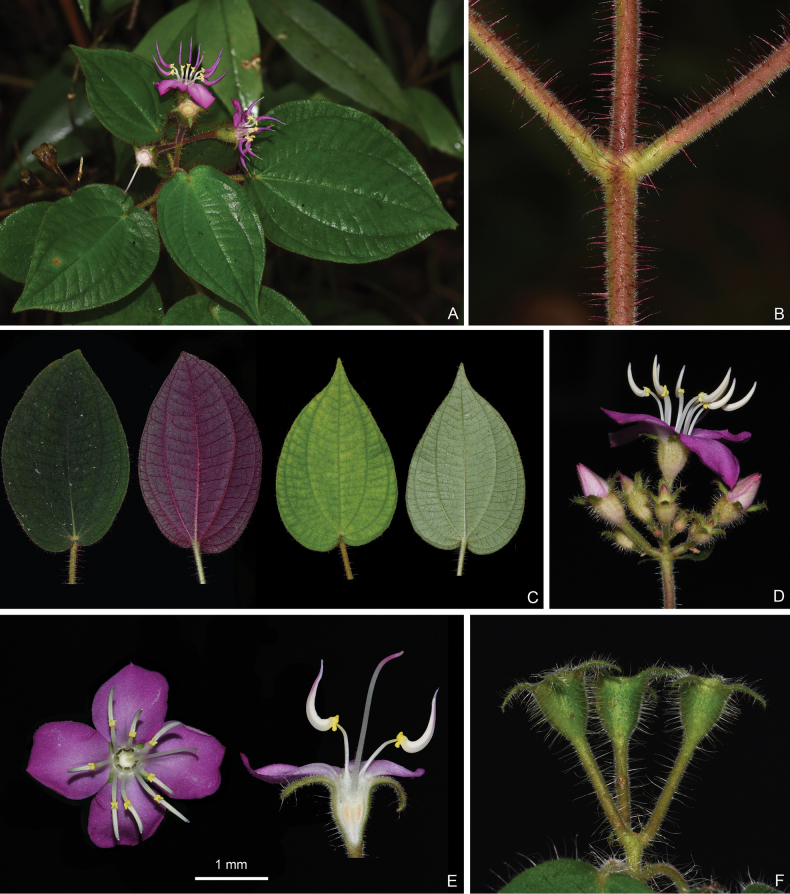
*Bredia fordii*. A. A flowering branch; B. Indumentum on a branchlet; C. Leaf morphology; D. An inflorescence; E. Top view (left) and longitudinal section (right) of a flower; F. Young capsules. Scale bar: 1 mm (E).

**Figure 7.**
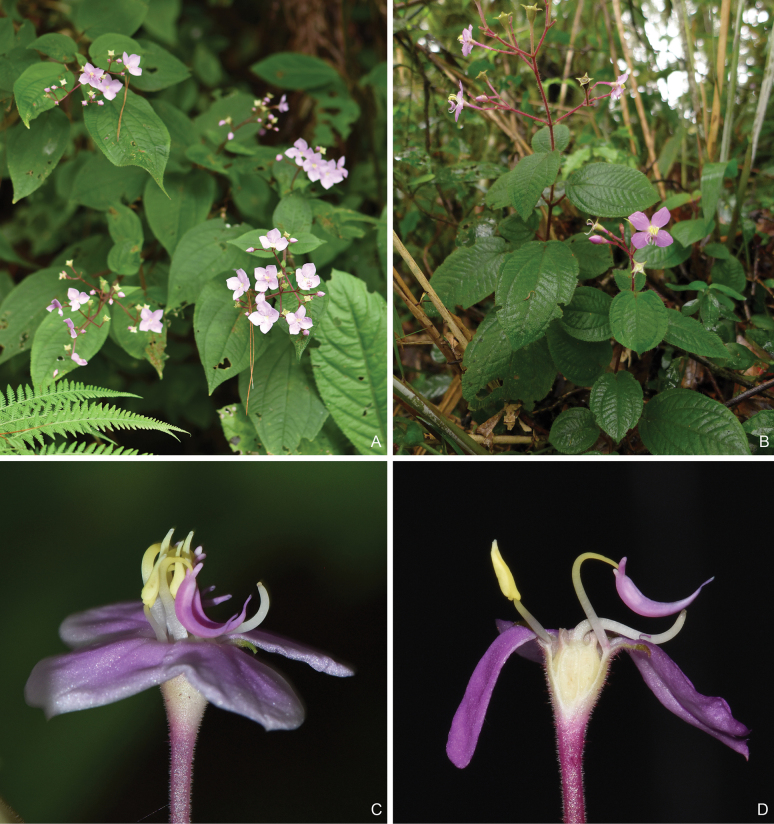
*Bredia hirsuta*, A and C from Okinawa, Japan (the type locality of *B. hirsuta*), B and D from Mt. Dulan, Taitung (the type locality of *B. dulanica*). A, B. Habit; C, D. Stamen morphology.

**Figure 8.**
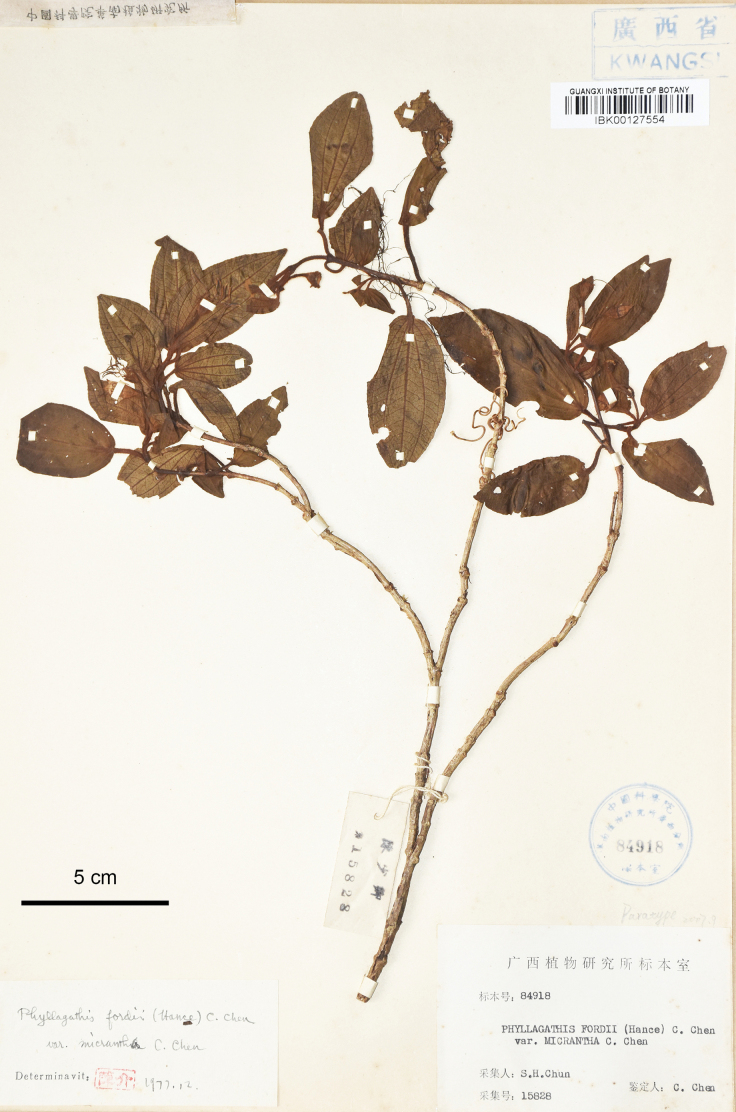
Holotype of *Bredia jiuwanshanensis*, S.H.Chun 15828 (IBK00127554). Scale bar: 5 cm.

**Figure 9.**
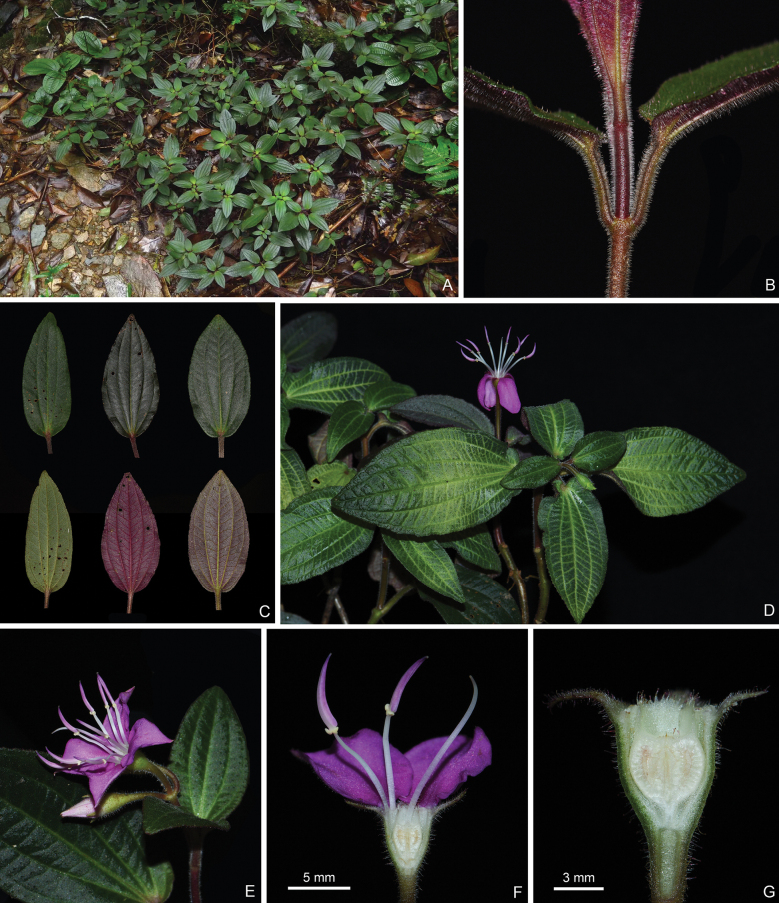
*Bredia jiuwanshanensis*, all from J.H.Dai and Y.Liu 880 (A, PE, SYS). A. Habit; B. Indumentum on a branchlet; C. Leaf morphology; D. A flowering branch; E. An inflorescence; F. Longitudinal section of a flower; G. Longitudinal section of a young capsule. Scale bars: 5 mm (F); 3 mm (G).

**Figure 10.**
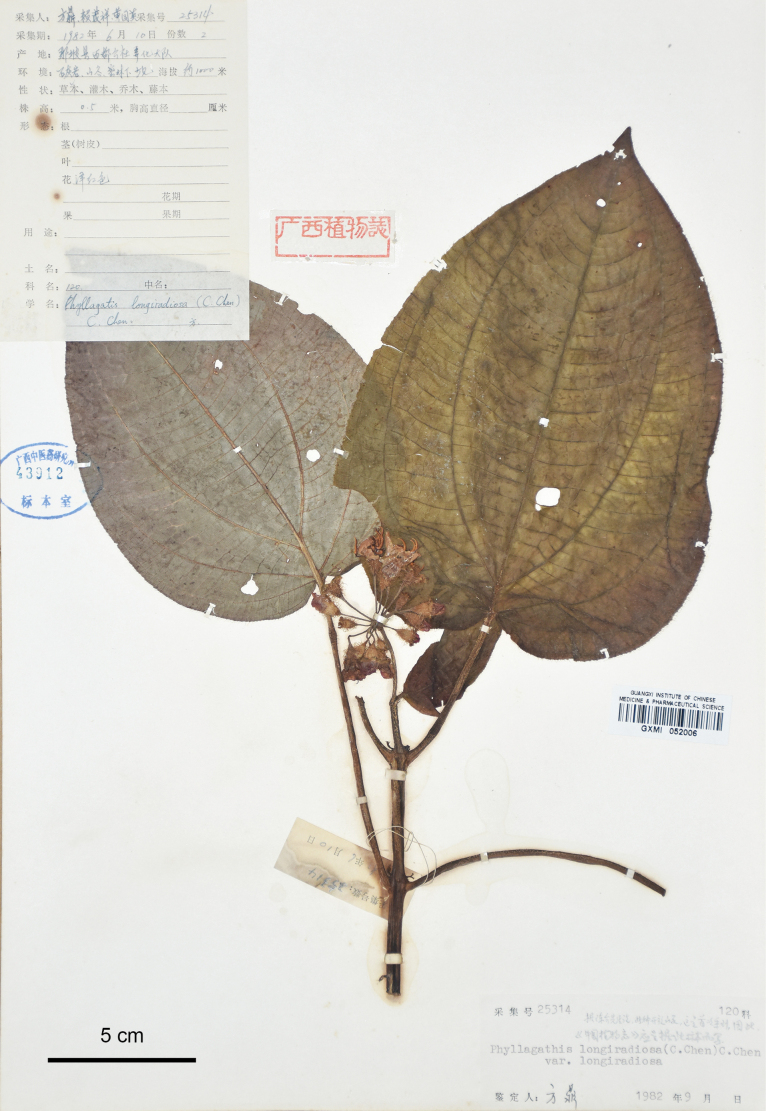
Holotype of *Bredia macrophylla*, D.Fang et al. 25314 (GXMI052006). Scale bar: 5 cm.

**Figure 11.**
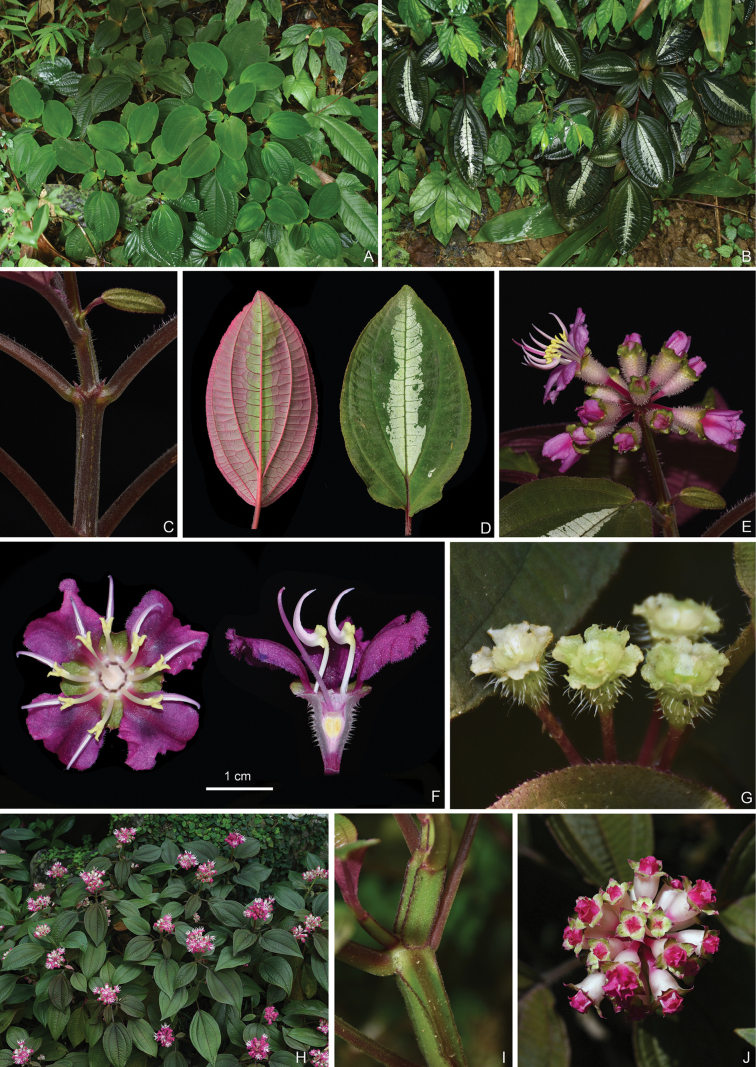
Bredia macrophylla var. macrophylla (A–G) and B. macrophylla var. pulchella (H–J). A, B, H. Habit; C, I. Branchlets; D. Leaf morphology; E, J. Inflorescences; F. Top view (left) and longitudinal section (right) of a flower; G. Young capsules. Photographs H and J by Hua-Fei Cen. Scale bar: 1 cm (F).

**Figure 12.**
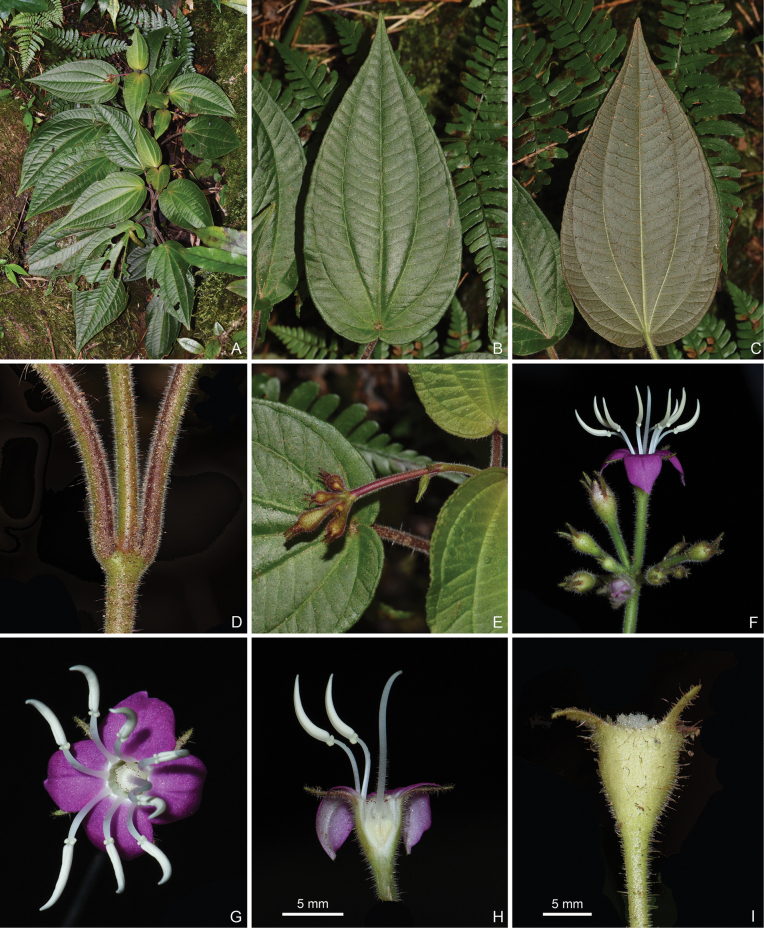
*Bredia micrantha*, all from J.H.Dai and Y.Liu 745 (A, PE, SYS). A. Habit; B, C. Adaxial and abaxial views of a leaf; D. Indumentum on a branchlet; E. Young inflorescence; F. Mature inflorescence; G. Top view of a flower; H. Longitudinal section of a flower; I. Lateral view of a young capsule. Scale bars: 5 mm (H, I).

**Figure 13.**
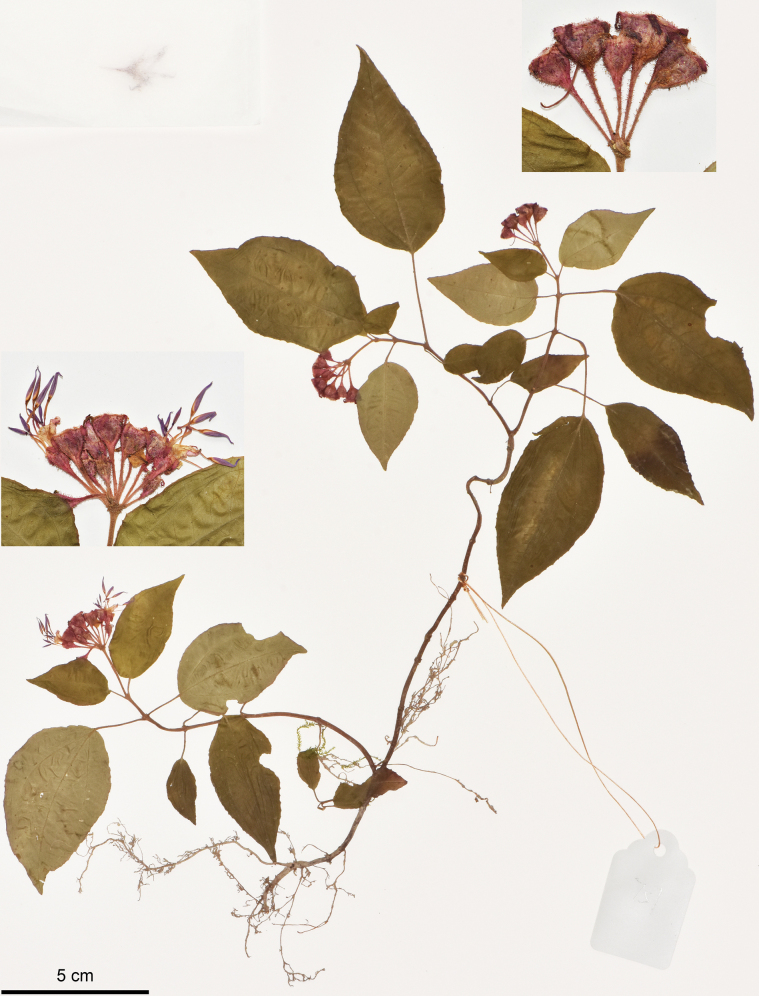
Holotype of *Bredia pingshanensis*, J.H.Dai and Y.Liu 757 (PE). The insets show details of flowers and young capsules. Scale bar: 5 cm.

**Figure 14.**
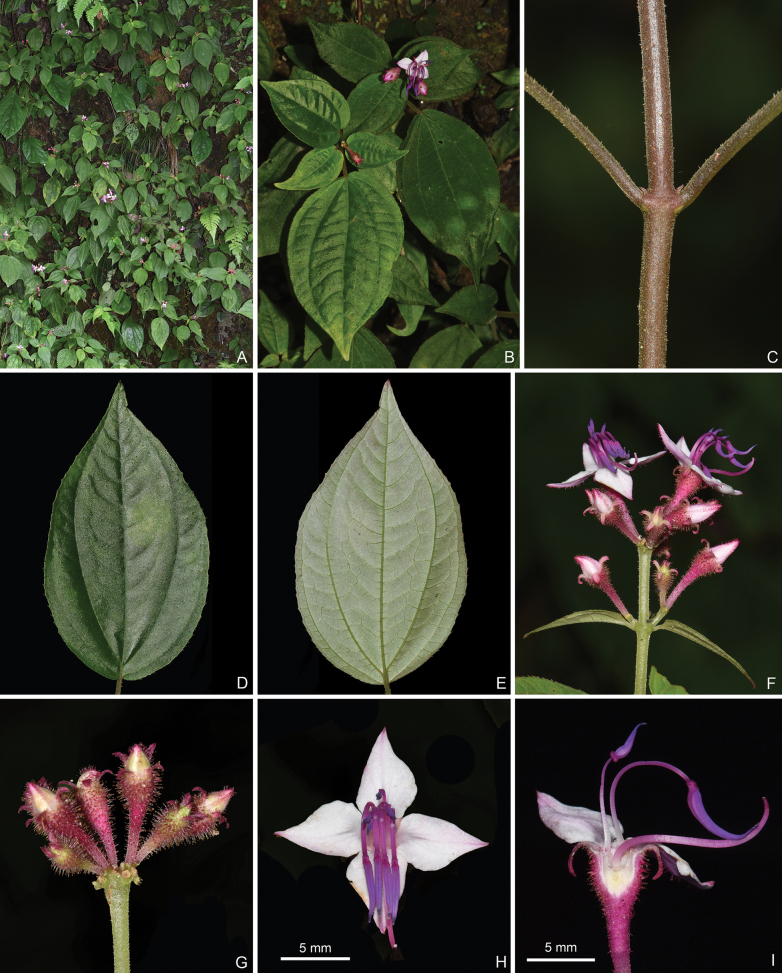
*Bredia pingshanensis*, all from J.H.Dai and Y.Liu 757 (A, PE, SYS). A. Habitat and habit; B. Flowering branches; C. Close-up of a branchlet; D, E. Adaxial and abaxial views of a leaf; F, G. Inflorescences; H. Top view of a flower; I. Longitudinal section of a flower. Scale bars: 5 mm (H, I).

**Figure 15.**
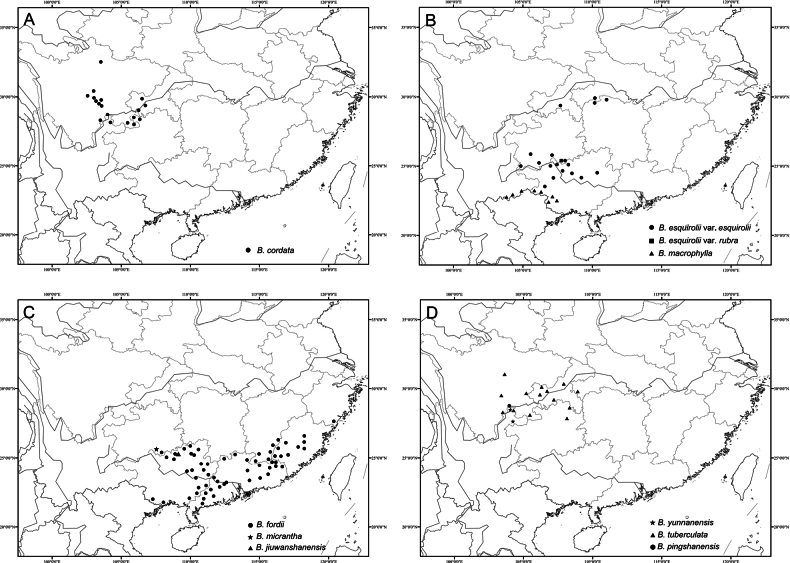
Distribution of the species discussed. A. *B. cordata*; B. *B. esquirolii* and *B. macrophylla*; C. *B. fordii*, *B. micrantha*, and *B. jiuwanshanensis*; D. *B. pingshanensis*, *B. yunnanensis*, and *B. tuberculata*.

Based on the findings of Dai et al. (2025), we present a taxonomic revision of some *Bredia* species, with the following updates: (1) *B. latisepala*, *B. longearistata*, and *B. longiradiosa* are synonymized under *B. esquirolii*; (2) *B. dulanica* C.L.Yeh, S.W.Chung & T.C.Hsu is incorporated within *B. hirsuta*; (3) two new combinations are established, alongside the formal recognition of three new species and one new variety (Suppl. material 1); (4) *Bredia bullata* S.Jin Zeng & N.H.Xia is regarded as a later homonym and therefore illegitimate name for *B. bullata* J.H.Dai & Ying Liu. As a result of these revisions, *Bredia* now comprises 28 species and three varieties. For the relevant taxa, we provided morphological descriptions, color plates (Figs 2–14), and updated distribution maps (Fig. 15), together with a key to all currently recognized species of *Bredia*.

## ﻿Methods

Morphological data were gathered through previous literature (Chen 1979; Chen 1984a, 1984b; Chen and Renner 2007; Yeh et al. 2008), examination of herbarium specimens (A, AU, BM, BNU, CDBI, CSFI, E, FJIDC, FJSI, GNNU, GXMG, GXMI, GZTM, HGAS, HIB, HITBC, IBK, IBSC, IFP, JIU, JXCM, KUN, LBG, MUCH, NAS, P, PE, QNUN, SYS, SZG, TAIF, WUK, XIN) and their high-resolution photographs, as well as observations of living plants both in the field and at the facilities of Sun Yat-sen University. Species distributions were mapped primarily based on herbarium records and supplemented by information from online databases such as Chinese Virtual Herbarium (https://www.cvh.ac.cn/), National Specimen Information Infrastructures (http://www.nsii.org.cn/), and the Plant Photo Bank of China (https://ppbc.iplant.cn/).

## ﻿Taxonomy

### ﻿ Bredia bullata

Taxon classificationPlantaeMyrtalesMelastomataceae

J.H.Dai & Ying Liu, PhytoKeys 195: 112. 2022.

A6BB49A5-999B-57BA-BE7D-D64A9B352C06

= Bredia bullata S.Jin Zeng & N.H.Xia, Phytotaxa 550(3): 282. 2022, nom. illeg. Type. China. Yunnan: Malipo County, Ba-bu Town, 1,221 m, 28 May 2020, S.J.Zeng 5017 (holotype: IBSC; isotypes: CANT, PE).

#### Type.

China • Yunnan: Malipo County, Ba-bu Town, Da-nong Village, 1,300 m, under forests, on limestone rocks, 30 May 2020, Y.Liu and J.H.Dai 849 (holotype: PE!; isotypes: A!, SYS!).

#### Notes.

*Bredia bullata* J.H.Dai & Ying Liu and *B. bullata* S.Jin Zeng & N.H.Xia were both described from the same population collected in Ba-bu Town, Malipo County, Yunnan Province. The former was published in May 2022 and the latter in June 2022. Accordingly, *B. bullata* S.Jin Zeng & N.H.Xia is regarded as an illegitimate later homonym of *B. bullata* J.H.Dai & Ying Liu.

### ﻿ Bredia cordata

Taxon classificationPlantaeMyrtalesMelastomataceae

H.L.Li, J. Arnold Arbor. 25: 24. 1944.

A8115916-4F95-5BF4-B1BE-E370EA4D5F2B

Figs 2, 15A

≡ Bredia esquirolii var. cordata (H.L.Li) C.Chen, Bull. Bot. Res., Harbin 4(3): 40. 1984.
= Phyllagathis fordii var. micrantha C.Chen, Bull. Bot. Res., Harbin 4(3): 50. 1984, p. p., quoad specim. T.C.Li 4756, F.T.Wang 23533, C.Y.Wu et al. 6307, C.Y.Wu 6325, Z.T.Guan 6154.
= Bredia esquirolii auct. non. (H.Lév.) Lauener, Chen in Fl. Reipubl. Popularis Sin. 53(1): 206; Chen et Renner in Fl. China 13: 375.

#### Type.

China • Sichuan: Ya-an, dense forest shade, 686 m, 30 Jul 1939, C.Y.Chiao 1205 [holotype: A! (A00071982)].

#### Description.

Shrubs 20–50 cm tall, branched. Stems erect, branched, terete, obtusely 4-sided, densely pubescent with 0.3–1 mm long multiseriate glandular hairs and puberulent with minute uniseriate (spreading or bent) hairs, rarely without multiseriate hairs. Leaves opposite, equal or unequal; petiole 1–8 cm long, pubescent and puberulent as the stem; leaf blade ovate, ovate-elliptic, or oblong-ovate, 2–14 × 1.5–8.2 cm, thin to thick papery, secondary veins 3 on each side of midvein, abaxial surface pale green to purplish, adaxial surface green to dark green, both surfaces puberulent with uniseriate hairs and sparsely strigose with multiseriate hairs or abaxially glabrescent and adaxially sparsely strigose, base cordate, margin serrulate or inconspicuously so with each tooth having a terminal seta, apex acuminate or short acuminate, rarely acute. Inflorescence a terminal cyme, rarely a cymose panicle; peduncle 1–3.5 cm long, indumentum same as the stem, 2–12-flowered. Flowers bisexual, radial but androecium slightly bilateral, 4-merous; pedicel 0.7–2 cm long, indumentum same as the stem; hypanthium bell- or funnel-shaped, 4–6 mm long, pubescent with glandular hairs, rarely only puberulent; calyx lobes linear-lanceolate, 3–5 mm long, pubescent with glandular hairs, rarely only puberulent; petals pink to purplish-red, ovate, 6–10 × ca. 6 mm, oblique, abaxially puberulent with minute uniseriate hairs or glabrescent, apex acute; stamens 8 in two whorls, often dimorphic, sometimes isomorphic, the outer whorl of the dimorphic stamens ca. 1.8 cm long, filaments ca. 10 mm long, anthers linear, ca. 7 mm long, curved, connective decurrent, slightly prolonged, the inner whorl of the dimorphic stamens or the isomorphic ca. 1.1 cm long, filaments ca. 6 mm, anthers lanceolate, slightly curved, ca. 5 mm long, forming two ventral lobes and a dorsal tubercule/short spur; ovary half inferior, locules 4, apex with a membranous crown, crown margin denticulate and ciliate with glandular hairs; style ca. 8–12 mm long, basally puberulent. Capsule ca. 6–9 × 5–7 mm, funnel-shaped, with enlarged apical crown; placentation axial, placentas non-thready. Seeds numerous, cuneate.

#### Phenology.

Flowering June to August, fruiting September to October.

#### Notes.

*Bredia cordata* can be readily distinguished from *B. esquirolii* by the leaf blade having 3 secondary veins (vs. 2) on each side of midvein, smooth petal margin (vs. undulate), linear-lanceolate calyx lobes (vs. broadly ovate to semiorbicular), and often dimorphic stamens (vs. isomorphic). It occurs in Sichuan, northern Guizhou, southern Chongqing, and northeastern Yunnan. Most populations have dimorphic stamens. However, both isomorphic and dimorphic morphs have been recorded in different populations and even within the same population in Sichuan and Chongqing, a rare occurrence in Melastomataceae.

#### Additional specimen examined.

**China. Chongqing Municipality**: • Beibei District, S.J.Wang 1231 (NAS), Sichuan-Guizhou Exped. 260 (PE), 759 (PE); • Jiangjin District, Z.Y.Liu 183157 (PE); • Banan District, Z.Y.Liu 180119 (PE). **Guizhou Province**: • Chishui County, M.C.Wang 520381150502008LY (GZTM), C.K.Liu CS9174 (MUCH); • Xishui County, Bijie Exped. 1695 (HGAS, KUN, PE). **Sichuan Province**: • Emeishan County, Z.W.Yao 2558 (KUN), T.C.Lee 4756 (KUN), F.T.Wang 23533 (WUK); • Gulin County, PE-Gulin Exped. 617 (PE); • Hongya County, Z.W.Wang 477 (CDBI), W.K.Bao 1241 (CDBI); • Jiajiang County, Y.Z.Tang et al. s.n. (CDBI); • Leshan County, Z.T.Guan 6154 (IBSC, KUN, PE); • Leibo County, Anonymous 574 (PE); • Pingshan County, Sichuan Econ. Pl. Exped. Yibin Division 753 (KUN); • Songpan County, W.P.Fang 6024 (PE); • Tianquan County, W.G.Hu and Z.He 11827 (PE); • Xuyong County, W.B.Ju and H.N.Deng HGX13070 (CDBI). **Yunnan Province**: • Suijiang County, B.X.Sun et al. 310 (PE), 534 (KUN); • Yanjin County, NE Yunnan Exped. 923 (KUN).

### ﻿ Bredia esquirolii

Taxon classificationPlantaeMyrtalesMelastomataceae

(H.Lév.) Lauener, Notes Roy. Bot. Gard. Edinburgh 31(3): 398. 1972.

1929F6AA-37DB-5FBA-AC95-17740A5D419D

Figs 3, 15B

≡ Barthea esquirolii H.Lév., Repert. Spec. Nov. Regni Veg. 11: 494. 1913 (Basionym). Type: China. Guizhou: Tchai-choui-ho, Jul 1909, Esquirol 1581 [holotype: E! (E00090793)].
= Barthea cavaleriei H.Lév., Repert. Spec. Nov. Regni Veg. 8: 61. 1910, p. p., excl. specim. Esquirol 215. Type: China. Guizhou: near Mou-you-sé, J.Cavalerie 1552 [lectotype, designated by Diels (1932): E! (E00090789)]. ≡ Fordiophyton cavaleriei (H.Lév.) Guillaumin, Bull. Soc. Bot. France 60: 275. 1913, p. p., excl. pl. Yunnan. ≡ Bredia cavaleriei (H.Lév.) Diels, Bot. Jahrb. Syst. 65(2–3): 110. 1932, hom. illeg., non H.Lév. & Vaniot (1906). ≡ Bredia longiradiosa C.Chen, Fl. Yunnan. 2: 105, 1979, nom. inval., p. p., quoad pl. Guizhou. Type: Based on Barthea cavaleriei H.Lév. ≡ Bredia longiradiosa (C.Chen) C.Chen ex Govaerts, World Checkl. Seed Pl. 2(1–2): 13. 1996, p. p., quoad typum. ≡ Phyllagathis longiradiosa C.Chen, Bull. Bot. Res., Harbin 4(3): 51. 1984, p. p., quoad pl. Guizhou. syn. nov.
= Phyllagathis longearistata C.Chen, Bull. Bot. Res., Harbin 4(3): 52. 1984. Type: China. Guangxi: Hechi, prope rivulos in convallibus montanis, 19 May 1928, L.H.Chun 91861 [holotype: IBK! (IBK00190677); isotype: IBK! (IBK00190678)]. ≡ Bredia longearistata (C.Chen) R.Zhou & Ying Liu, PhytoKeys 127: 145. 2019. syn. nov.
= Phyllagathis latisepala C.Chen, Bull. Bot. Res., Harbin 4(3): 53. 1984. Type: China. Hubei: Hefeng, ad pedes montis calcareo, 18 Sept 1958, H.J.Li 6451 [holotype: IBSC! (IBSC0003996); isotypes: PE! (PE00025692), HIB (HIB0060155)]. ≡ Bredia latisepala (C.Chen) R.Zhou & Ying Liu, PhytoKeys 127: 145. 2019. syn. nov.

#### Description.

Herbs or shrubs, 20–40 cm tall. Stems prostrate in lower parts and ascending in the upper parts, terete, sparsely villous with spreading multiseriate hairs and puberulent with bent uniseriate hairs when young, sometimes glabrescent. Leaves opposite; petiole 2–8 cm long, indumentum same as branchlets; leaf blade ovate to oblong ovate, 3.5–9 × 1.5–6 cm, submembranous, secondary veins 2 on each side of midvein, adaxial surface green to purplish dark green, abaxial surface pale green to purplish-red, with bent uniseriate hairs and sparse multiseriate setas when young on both sides, with minute yellowish glandular hairs on the abaxial surface, base subcordate to obtuse, margin inconspicuously denticulate with each tooth having a terminal seta, apex acute or short acuminate. Inflorescence terminal, umbellate, rarely cymose paniculate, 3–11-flowered; peduncle 1.5–5 cm long. Flowers bisexual, radial but androecium slightly bilateral, 4-merous; pedicel 1–2 cm long; hypanthium yellowish-green, funnel-shaped, ca. 5 mm long, villous with multiseriate hairs with inflate bases or only puberulent; calyx lobes 4, broadly ovate to semiorbicular; petals 4, pink to purplish, ovate, ca. 6 × 7 mm, petal margin undulate, apex oblique; stamens 8 in two whorls, isomorphic, equal in length, filaments ca. 5 mm long, bent with the anthers to one side of the flower, anthers lanceolate, slightly curved, ca. 7 mm long, white, connectives forming 2 ventral lobes and a dorsal short spur, white or light purple; ovary ca. 3 mm long (crown excluded), half inferior, locules 4, apex with membranous crown, crown margin denticulate; style ca. 1 cm long, basally puberulous. Capsule funnel-shaped, with enlarged apical crown; placentation axial, placentas non-thready. Seeds numerous, cuneate.

#### Phenology.

Flowering April to July, fruiting May to August.

#### Distribution.

*Bredia esquirolii* is known from Guangxi, southern Guizhou, Hunan, Chongqing, and Hubei, China, growing in karst or non-karst habitats in forest or along forest margin at 250–1,200 m.

#### Additional specimen examined.

**China. Chongqing Municipality**: • Wulun County, Z.Y.Liu 181907 (PE). **Guangxi Province**: • Bama County, W.B.Xu and Y.Liu 09344 (IBK); • Debao County, Debao Exped. 451024170219012LY (IBK); • Fengshan County, Y.D.Peng et al. 451223121026092LY (GXMG); • Hechi City, Mulun Exped. M0220 (PE), L.H.Chun 92034 (IBK), Huanjiang Exped. 451226130314019LY (GXMG, IBK), 451226130723003LY (GXMG, IBK), Hechi Exped. 4-4-465 (GXMI), W.B.Xu and B.Pan 09527 (IBK), W.B.Xu 091711 (IBK), Y.Liu 496 (SYS), 498 (SYS), 859 (SYS); • Lipu County, Lipu Exped. 450331180823035LY (IBK), 450331170710033LY (IBK), 450331180520007LY (IBK); • Liujiang County, Liujiang Exped. 450221200728017LY (IBK), 450221200730042LY (IBK), 450221200731016LY (IBK), 450221190718010LY (IBK), 450221190403042LY (IBK), 450221180810019LY (IBK); • Nandan County, Nandan Exped. 4-5-1005 (GXMI), Nandan Exped. 451221180526064LY (GXMG), 451221180525032LY (GXMG), 451221190705036LY (GXMG); Tian’e County, Beijing Team 891333 (PE), 891340 (PE), D.Y.Liu 58480 (GXMI). **Guizhou Province**: • Without precise location, Cavalerie 3638 (P), Cavalerie 2015 (P), Esquirol 3148 (P); • Libo County, Q.R.Liu 2018080412 (BNU), Y.Liu 744 (SYS), 875 (SYS); • Xingyi County, Cavalerie 1917 (P), Esquirol 4571 (P), C.Y.Deng 2021051961 (XIN); • Wangmo County, Y.Liu 912 (SYS). **Hunan Province**: • Baojing County, D.G.Zhang 0905626 (JIU), X.J.Su and H.B.Liu 433125D00031114003 (JIU); • Sangzhi County, Sangzhi Forestry Institute 1150 (KUN), Anonymous 614 (PE), Beijing Team 4043 (PE), 2931 (PE), 2999 (PE), Y.Liu 557 (SYS); • Shimen County, Hupingshan Exped. 0880 (PE).

### ﻿ Bredia esquirolii var. rubra

Taxon classificationPlantaeMyrtalesMelastomataceae

J.H.Dai & Ying Liu var. nov.

EF5B44FF-C3A7-581F-B997-00AEA15CBA5B

urn:lsid:ipni.org:names:77371611-1

Figs 4, 5, 15B

#### Type.

China • Guizhou: Libo County, Maolan Town, Pang-xie-gou, ca. 500 m, 3 Apr 2022, *Y.Liu and J.H.Dai 864* (holotype: PE!; isotypes: SYS!).

#### Diagnosis.

Bredia esquirolii var. rubra differs from B. esquirolii var. esquirolii in having dense reddish multiseriate hairs on the stems, leaves, and inflorescences.

#### Phenology.

Flowering in late March to April, fruiting May to June.

#### Etymology.

The specific epithet refers to the reddish hairs on many parts of the plant.

#### Distribution.

This variety is currently only known from Libo, Guizhou, China, growing in moist places under forests.

#### Additional specimen examined.

**China. Guizhou Province**: • Libo County, J.J.Li 20183113 (QNUN), M.Hu QNSY20182148 (QNUN), M.J.He 20185348 (QNUN), S.H.Yang 20183109 (QNUN), M.Deng 20188529 (QNUN), X.J.Ma 20188464 (QNUN), J.W.Zhang 20181054 (QNUN), Q.L.Yang 201812072 (QNUN), D.Y.Cen 20188495 (QNUN), L.Wu and F.L.Chen 6426 (CSFI), X.M.Wang 0351 (HGAS).

### ﻿ Bredia fordii

Taxon classificationPlantaeMyrtalesMelastomataceae

(Hance) Diels, Bot. Jahrb. Syst. 65(2–3): 110. 1932.

A15DC989-9D7C-5400-A911-C4F06606CACB

Figs 6, 15C

≡ Otanthera fordii Hance, J. Bot. 19: 47. 1881 (Basionym). Type: China. Hong Kong, Jul 1880, C.Ford. herb no. 21099 [lectotype, designated by Zhou et al. (2019c): BM! (BM000629024); isolectotype BM! (BM000629025)]. ≡ Phyllagathis fordii (Hance) C.Chen, Bull. Bot. Res., Harbin 4(3): 50. 1984.
= Bredia sepalosa Diels, Bot. Jahrb. Syst. 65(2–3): 109. 1932. Type: China. Guangxi: Yaoshan, 1928, S.S.Sin & K.K.Whang 648 [lectotype, designated by Zhou et al. (2019c): IBSC! (IBSC0003942)].
= Bredia tuberculata auct. non. (Guillaumin) Diels, H.L.Li, J. Arnold Arbor. 25(1): 23. 1944.
= Phyllagathis fordii var. micrantha C.Chen, Bull. Bot. Res., Harbin 4(3): 50. 1984, p. p., quoad specim. Z.C.Chen 51718, 51752, S.L.Yu 900451, H.F.Qin and Z.T.Li 71086.

#### Notes.

*Bredia fordii* is the most widely distributed species in the genus, found in Guangdong, Guangxi, Fujian, Jiangxi, southern Guizhou (Libo County), and southern Zhejiang (Pingyang County). The plant exhibits considerable variation in posture, plant height, leaf size and color, and density of the spreading multiseriate hairs on the stem, among populations from different localities. Nevertheless, *B. fordii* can be readily distinguished by the 3–5 mm long, spreading multiseriate hairs on many parts of the plant (stem, leaves, inflorescence, hypanthium, calyx lobes), as well as its isomorphic stamens with geniculate anthers, often purplish anther sacks, and yellow anther appendages.

#### Additional specimen examined.

**China. Fujian Province**: • Changting County, Y.Ling 5133 (PE); • Jiangle County, Longxishan Exped. 1607 (PE); • Longyan County, D.S.Wang s.n. (PE), 1255 (AU, PE), H.B.Chen 1230 (FJSI); • Mingxi County, Mingxi Exped. 5097 (NAS), L.G.Ling 331 (PE); • Shanghang County, L.G.Lin 6918, 7024 (PE), Y.Ling 5420, 5421 (PE), Anonymous 634 (FJIDC), Y.T.Zhang 82107 (FJSI), Meihuashan Exped. 205 (FJSI); • Shunchang County, G.S.He 326 (FJSI, PE), M.S.Li and Z.Y.Li 4712 (PE), 4790 (FJSI, PE); • Yong’an County, Y.Ling 3004 (PE); • Youxi County, Anonymous 588 (FJIDC). **Guangdong Province**: • Boluo County, Guangdong-78 6201 (IBSC), N.K.Chun 41030 (IBSC), 41387 (IBSC); • Dapu County, L.Tang 5322 (IBSC); • Deqing County, Y.G.Liu 962 (HHBG, HIB, NAS); • Fengkai County, B.H.Chen 1440 (IBSC), G.L.Shi 14792 (IBSC), 14805 (IBSC), L.H.Chiu 50082 (IBSC), Fengkai Exped. 4748 (IBSC), 5022 (IBSC), G.Q.Ding 6028 (IBSC), S.H.Chun 18451 (IBSC); • Gaoyao County, G.Q.Ding and G.L.Shi 1587 (IBSC), Y.G.Liu 1957 (IBSC); • Gaozhou County, P.C.Zhou 10279 (IBSC); • Heping County, Heping Exped. 754 (IBSC); • Jiaoling County, L.Tang 4593 (IBSC), 4832 (IBSC); • Lechang County, S.Wang 143183 (IBSC), N.K.Chun 42395 (IBSC); • Lianxian County (now Lianzhou), B.Y.Liu 2036 (IBSC); • Luoding County, N.Liu et al. 2316 (IBSC); • Maoming County, L.Tang 2351 (IBSC), 2386 (IBSC), 2371 (IBSC); • Meixian County, W.T.Tsang 21515 (IBSC); • Pingyuan County, L.Tang 4040 (IBSC), 4302 (IBSC); • Shixing County, G.Yao 271 (IBSC), Shixing Exped. 78 (IBSC); • Wuhua County, S.C.Lee 201649 (IBSC); • Xinfeng County, L.Tang 8020 (AU, IBK, IBSC, KUN, NAS, PE, WUK), H.G.Ye 1050 (IBSC), 1192 (IBSC); • Xingning County, S.C.Lee 201828 (IBSC); • Xinyi County, C.Wang 30902 (IBK, IBSC, IFP, KUN, PE, WUK), 31778 (IBK, IBSC), 37923 (IBK, IBSC), C.M.Tan and L.P.Zhang Xinyi-024 (JJF); • Yangchun County, H.G.Ye and N.Liu 1714 (IBSC), 1739 (IBSC), Yunkai Exped. 162 (IBSC), H.G.Ye et al. 5676 (IBSC), 6694 (IBSC); • Yunfu County, L.Tang 10132 (IBSC), C.Wang 37291(IBSC); • Zhaoqing County, G.L.Shi 12990 (IBSC), 14040 (IBSC); • Zijin County, Z.F.Wei 120977 (IBSC). **Guangxi Province**: • Bobai County, M.L.Zhang 16443 (PE); • Cangwu County, S.H.Chun 9979 (IBK, IBSC, KUN); • Guiping County, Guiping Exped. 450881171030088LY (GXMG), 450881190727027LY (GXMG), 450881181021014LY (GXMG); • Jinxiu County, Y.Liu et al. H0428 (PE), S.J.Zhang 10-4302 (HIB), Y.K.Li 400580 (IBK, IBSC), Y.C.Chen 1609 (IBK), Dayaoshan Exped. 10123 (IBK, IBSC), 10404 (IBK), 11319 (IBK, IBSC), 12635 (IBK, IBSC), 12795 (IBK, IBSC), 810358 (IBK), 810437 (GXMI, IBK), 811024 (IBK); • Lingchuan County, Lingchuan Exped. 450323130808019LY (GXMG, IBK); • Lingui County, C.F.Liang 30776 (KUN); • Longsheng County, Q.H.Lu and Y.Z.Wei 20487 (IBK), Z.T.Li and H.F.Qin 71086 (IBK); • Luocheng County, Y.Qin et al. JWS201027027 (IBK), Z.R.Liu et al. JWS201030002 (IBK), Luocheng Exped. 451225130728012LY (GXMG, IBK), Beijing Exped. 895753 (PE), S.H.Chun 14955 (IBK, IBSC, IFP, KUN, LBG, NAS, PE); • Ningming County, Z.Q.Zhang 12878 (IBSC, KUN); • Pingle County, Pingle Exped. 450330180807069LY (IBK), 450330180912007LY (IBK), 450330180808021LY (IBK), Y.K.Li 401943 (IBK, IBSC); • Pingnan County, J.X.Zhong 84778 (IBK); • Rongxian County, D.Fang 11814 (GXMI, PE), S.H.Chun 9810 (IBK, KUN, LBG, PE), Rongxian Exped. 450921180613029LY (GXMG), Z.Y.Wei and D.M.Lei 40212 (IBK), 40262 (IBK); • Rongshui County, S.H.Chun 15635 (HITBC, IBK, IBSC, KUN, NAS, WUK), S.H.Chun 15636 (HITBC, IBK, IBSC, PE), Beijing Exped. 897339 (PE), 897364 (PE); • Sanjiang County, H.J.Wu 450226160805012LY (GXMG); • Shangsi County, Beijing Youth Exped. 609, 612 (PE), W.T.Tsang 22563 (IBK, WUK), 22457 (IBK); • Xiangzhou County, C.Wang 40241 (IBSC, PE); • Xing’an County, Guangxi Exped. 898 (PE), G.Z.Li 15278 (PE), J.X.Han 092 (PE), G.Z.Li 15208 (PE), Xing’an Exped. 450325130715018LY (GXMG), Z.Z.Chen 51718 (IBK, IBSC, KUN), 51752 (IBK), S.L.Yu 900451 (IBK), G.Z.Li 11987 (IBK), 12897 (IBK); • Yongfu County, Yongfu Exped. 450326130803095LY (GXMG, IBK); • Zhaoping County, Qichong Exped. QC211 (IBK), Y.Qin et al. QC865 (IBK), X.K.Huang et al. QC1578 (IBK), S.Y.Nong et al. QC1684 (IBK), G.Xie et al. QC2086 (IBK); • Ziyuan County, Ziyuan Exped. 450329160816002LY (GXMG, IBK), G.Z.Li and Z.X.Liao 10133 (IBK, SZG). **Guizhou Province**: • Libo County, Y. He 2-295 (PE), Libo Exped. 1145 (HGAS, KUN, PE). **Jiangxi Province**: • Huichang County, Q.M.Hu 2857 (LBG), 3101 (LBG); • Longnan County, Jiangxi Exped. 426 (PE), 236 Task Group 1289 (PE); • Ruijin County, Q.M.Hu 4339 (LBG, PE); • Xinfeng County, Z.B.Tang T180718201 (GNNU), R.L.Liu et al. L180719055 (GNNU), L180719054 (GNNU); • Xunwu County, J.S.Yue et al. 1957 (NSA, PE), Z.B.Tang 170823168 (GNNU), 170821068 (GNNU), L.Cao et al. 360734140414355LY (JXCM), Y.Lin 15081 (LBG), 15150 (LBG), Q.M.Hu and Q.H.Li 1375 (LBG). **Zhejiang Province**: • Pingyang County, Anonymous 3163 (HHBG), Anonymous 24895 (NAS).

### ﻿ Bredia hirsuta

Taxon classificationPlantaeMyrtalesMelastomataceae

Blume, Mus. Bot. 1(2): 24. f. IV. 1849.

5DE55352-D48B-5AE1-993D-B33CE221C5CC

Fig. 7

= Bredia dulanica C.L.Yeh, S.W.Chung & T.C.Hsu, Edinburgh J. Bot. 65(3): 395 (398, fig. 2; 402, fig. 4A, B). 2008. syn. nov. Type: China. Taiwan Province: Taitung, Mt. Dulan, on the ridge of a mountain, 1000–1200 m, 14 Oct 2007, S.W.Chung, T.C.Hsu & C.R.Yeh 16 [holotype: TAIF! (TAIF348619); isotypes: TAIF! (TAIF348620, TAIF348621, TAIF348622)].

#### Type.

Japan • K. Ito s.n. [lectotype designated by Zhou et al. (2019c): L! (L0170980)].

#### Notes.

*Bredia dulanica* was described based on specimens collected from Mt. Dulan, southeastern Taiwan (Yeh et al. 2008). Yeh et al. (2008) suggested that it is closely related to *B. hirsuta*, a species found in Taiwan and the Ryukyu Islands, but can be distinguished by the rod-shaped ventral appendages and the calyx lobes with red strigose hairs abaxially. However, the accession from Mt. Dulan (LY 565) is deeply nested within *B. hirsuta* in the species tree based on single-copy orthologs (Fig. 1). A comparison of living plants of *B. dulanica* and *B. hirsuta* from Taiwan and Okinawa revealed no obvious difference in stamen morphology and hairiness (Fig. 7). Based on the above findings, we consider *B. dulanica* to be a synonym of *B. hirsuta*.

#### Additional specimen examined.

**China. Taiwan Province**: • Taidong County, S.M.Liu et al. 507 (PE), S.W.Chung 8254 (TAIF), 8249 (TAIF).

### ﻿ Bredia jiuwanshanensis

Taxon classificationPlantaeMyrtalesMelastomataceae

J.H.Dai & Ying Liu sp. nov.

4F483294-3F89-595D-831A-9B5434EF3BE2

urn:lsid:ipni.org:names:77371612-1

Figs 8, 9, 15C

= Phyllagathis fordii var. micrantha C.Chen, Bull. Bot. Res., Harbin 4(3): 50. 1984, p. p., quoad specim. S.H.Chun 15828.

#### Type.

China • Guangxi: Da-miao-shan County (now Rongshui County), Jiu-wan-shan, Shuang-he-gou, 750–1,200 m, 26 Jul 1958, *S.H.Chun 15828* [holotype: IBK! (IBK00127554); isotypes: HITBC! (HITBC011254), IBSC! (IBSC0223746), KUN! (KUN0156146), PE! (PE00782743)].

#### Diagnosis.

Most closely resembles *B. micrantha* in the dense, spreading, uniseriate hairs on the stems, bending young inflorescence, and isomorphic stamens, but differs in posture (multi-branched vs. few branched), smaller (1.5–7 × 0.7–3.8 cm vs. 3.5–13 × 1.7–6.3 cm), thick papery (vs. submembranous to thin papery), elliptic to narrowly elliptic leaf blade (vs. more or less ovate) with obtuse or rounded base (vs. cordate), and acute apex (vs. acuminate), and purple anthers and yellow connectives at anther base (vs. both cream).

#### Description.

Shrubs to 20 cm tall. Stems prostrate in lower parts and ascending/erect in upper parts, many-branched, terete; branchlets densely pubescent with 0.5 mm long, spreading, uniseriate hairs and multiseriate glandular hairs. Leaves opposite, equal to unequal; petiole 0.4–2.2 cm long, densely pubescent as branchlets; leaf blade elliptic, oblong-elliptic, or narrowly elliptic, 1.5–7 × 0.7–3.8 cm, thick papery, secondary veins 3 on each side of midvein, adaxial surface green to purplish dark green, puberulent with bent uniseriate hairs and ca. 0.2 mm long multiseriate setas, abaxial surface purplish-red, pubescent as branchlets, densely so along veins, base rounded, rarely truncate, margin ciliate and inconspicuously serrulate with each tooth having a terminal seta, apex acute. Inflorescence a terminal cyme, bending downwards when young, 1–3-flowered; peduncle 0.5–1.5 cm long, densely pubescent as branchlets. Flowers bisexual, radial but androecium slightly bilateral, 4-merous, pedicle, hypanthium and calyx lobes densely pubescent with 0.3–1 mm long hairs; pedicel 0.5–1.2 cm long; hypanthium yellowish-green, funnel-shaped, 5–6 × 3–4 mm; calyx lobes 4, narrowly triangular, 2–3 × 0.5 mm; petals 4, purplish-red, ovate, ca. 9 × 7 mm, puberulent with uniseriate hairs at the margin and along midvein on the abaxial surface, apex oblique, short acuminate; stamens 8 in two whorls, isomorphic, equal in length, filaments ca. 7 mm long, bent with the anthers to one side of the flower, anthers lanceolate, slightly curved, ca. 6 mm long, purplish-pink, connective forming 2 yellowish cream ventral lobes and a dorsal short spur of the same color; ovary ca. 4 mm long, 2/3 as long as the hypanthium (crown excluded), half inferior, locules 4, apex with membranous crown, crown margin ciliate with red glandular hairs; style ca. 1.4 cm long, basally puberulous. Capsule ca. 7 × 6 mm, funnel-shaped, with enlarged apical crown; placentation axial, placentas non-thready. Seeds numerous, cuneate.

#### Phenology.

Flowering late July to August, fruiting September.

#### Etymology.

The specific epithet refers to the type locality of the new species, Jiu-wan-shan Nature Reserve.

#### Distribution.

*Bredia jiuwanshanensis* is only known from Jiu-wan-shan Nature Reserve in Rongshui County, northern Guangxi, China, occupying moist soil slopes in forests or along forest margin.

#### Additional specimen examined.

**China. Guangxi Province**: • Rongshui County, Jiu-wan-shan Nature Reserve, Yang-mei-ao, Jiu-ren station, Bai-yan-shan, along forest margin and on soil slopes in forests, 1,400 m, 24 Jul 2021, J.H.Dai and Y.Liu 880 (A, PE, SYS).

### ﻿ Bredia macrophylla

Taxon classificationPlantaeMyrtalesMelastomataceae

J.H.Dai & Ying Liu sp. nov.

48F49EC5-8B3B-55D5-9072-AAF97A56FFD8

urn:lsid:ipni.org:names:77371613-1

Figs 10, 11, 15B

= Bredia longiradiosa C.Chen, Fl. Yunnan. 2: 105, 1979, nom. inval., p. p., excl. pl. Guizhou.
= Phyllagathis longiradiosa C.Chen, Bull. Bot. Res., Harbin 4(3): 51. 1984, p. p., excl. pl. Guizhou.

#### Type.

China • Guangxi: Napo County, Baidu Town, Nonghua Village, ca. 1,000 m, 10 Jun 1982, *D.Fang et al. 25314* [holotype: GXMI! (GXMI052006); isotype: GXMI! (GXMI052007)].

#### Diagnosis.

Most closely resembles *B. esquirolii* in habitat preference, leaf shape, umbellate inflorescence, villous hypanthium, broadly ovate to semiorbicular calyx lobes, and isomorphic stamens, but is readily distinguished by larger plant size (30–100 cm vs. 20–40 cm tall), larger leaves (7–23 × 5.5–13 cm vs. 3.5–9 × 1.5–6 cm), the lack of yellow glandular hairs on the abaxial leaf surface, geniculate anthers (vs. slightly curved), and yellow connectives (vs. white to light purple).

#### Description.

Herbs or shrubs, 30–100 cm tall. Stems erect, sometimes prostrate in lower parts, terete or 4-sided; branchlets near succulent, sparsely villous with multiseriate hairs or glabrescent, sparsely puberulent with bent uniseriate hairs. Leaves opposite; petiole 3–12 cm long, indumentum same as branchlets; leaf blade broadly ovate to subelliptic, 7–23 × 5.5–13 cm, submembranous to papery, secondary veins 3 on each side of midvein, adaxial surface green to purplish dark green, abaxial surface pale green to purplish-red, with bent uniseriate hairs and sparse multiseriate setas when young on both sides, base cordate to obtuse, margin denticulate to subentire and ciliate, apex short acuminate. Inflorescence terminal, umbellate, rarely cymose paniculate, 3–20-flowered; peduncle 2–5 cm long. Flowers bisexual, radial but androecium slightly bilateral, 4-merous; pedicel 0.5–1.2 cm long; hypanthium yellowish-green, funnel-shaped, ca. 1 cm long, villous with multiseriate hairs with inflate bases; calyx lobes 4, broadly ovate to semiorbicular; petals 4, purplish-red, ovate, ca. 7 × 9 mm, petal margin undulate, apex oblique; stamens 8 in two whorls, isomorphic, equal in length, filaments ca. 7 mm long, bent with the anthers to one side of the flower, anthers lanceolate, geniculate, ca. 10 mm long, purplish-pink, connective forming 2 yellow ventral lobes and a dorsal short spur of the same color; ovary ca. 5 mm long (crown excluded), half inferior, locules 4, apex with membranous crown, crown margin denticulate; style ca. 1.5 cm long, basally puberulous. Capsule funnel-shaped, with enlarged apical crown; placentation axial, placentas non-thready. Seeds numerous, cuneate.

#### Phenology.

Flowering April to June, fruiting July to August.

#### Etymology.

The specific epithet refers to the large leaves of the species.

#### Distribution.

*Bredia macrophylla* is known from limestone areas in southwestern Guangxi and southeastern Yunnan, China, occupying moist habitats in forest or at forest margin at 180–1,200 m.

#### Additional specimen examined.

**China. Guangxi Province**: • Chongzuo City, Y.D.Peng et al. 451402150915003LY (GXMG); • Daxin County, B.Y.Huang and R.C.Wei 451424150411033LY (GXMG); • Jingxi County, H.Z.Lv et al. 451025130315051LY (GXMG); • Longzhou County, CHN Herb. Guangxi Exped. 2760 (PE), P.X.Tan 57403 (IBSC), H.C.Li 40280 (IBK, IBSC), S.C.Chen 13294 (KUN), Deng et al. GXIBDT011B03 (KUN), Y.Liu 484 (SYS); • Napo County, CHN Herb. Guangxi Exped. 1615 (PE), 4148 (PE), D.Fang et al. 25032 (PE), S.P.Ko 55878 (IBSC, PE), D.X.Nong et al. 451026141012030LY (GXMG), 451026150528015LY (GXMG). **Yunnan Province**: • Hekou County, Y.M.Sui et al. 20907 (KUN), Y.Liu 730 (SYS); • Maguan County, C.J.Zhao 307 (HITB); • Malipo County, C.W.Wang 86169 (PE).

### ﻿ Bredia macrophylla var. pulchella

Taxon classificationPlantaeMyrtalesMelastomataceae

(C.Chen) J.H.Dai & Ying Liu comb. nov.

18D67109-AF88-5DF2-A8B8-83D7EC93BA20

urn:lsid:ipni.org:names:77371614-1

≡ Phyllagathis longiradiosa var. pulchella C.Chen, Bull. Bot. Res., Harbin 4(3): 52. 1984 (Basionym). Type: China. Guangxi: Longjin, 4 May 1959, F.F.Huang 3596 (holotype: GXMI! [GXMI050237]). ≡ Bredia longiradiosa var. pulchella (C.Chen) R.Zhou & Ying Liu, PhytoKeys 127: 146. 2019.

#### Notes.

Bredia macrophylla var. pulchella differs from B. macrophylla var. macrophylla in the stem, leaf petioles, and hypanthium only puberulent (vs. villous with multiseriate hairs), without multiseriate hairs with inflate bases.

#### Additional specimen examined.

**China. Guangxi Province**: • Daxin County, CHN Herb. Guangxi Exped. 0390 (PE), J.J.Wang 04220 (GXMI); • Jingxi County, H.Wang 6676 (PE); • Longzhou County, CHN Herb. Guangxi Exped. 0582 (PE), 0984 (PE), S.K.Li 200501 (IBK, IBSC), P.X.Tan 57212 (IBSC), S.C.Chen 13933 (HIBT, IBK, IBSC), Nonggang Exped. 10262 (IBK), 20407 (GXMI, IBK), Y.Liu and Y.S.Huang Y2372 (IBK), F.S.Huang 3596 (GXMI), J.Y.Luo and J.X.Ling 76256 (GXMI), Longzhou Med. Exped. 0331 (GXMI).

### ﻿ Bredia micrantha

Taxon classificationPlantaeMyrtalesMelastomataceae

(C.Chen) J.H.Dai & Ying Liu, comb. et stat. nov.

0339FB10-412C-5684-A831-8DA22982C863

urn:lsid:ipni.org:names:77371615-1

Figs 12, 15C

≡ Phyllagathis fordii var. micrantha C.Chen, Bull. Bot. Res., Harbin 4(3): 50. 1984 (Basionym), p. p., quoad typum. Type: China. Guizhou: Dushan, in convallibus montanis, 600 m, 22 Aug 1930, Y.Tsiang 6563 [holotype: IBSC! (IBSC0003995); isotypes: NAS! (NAS00052126), PE! (PE00782806, PE00782810)]. ≡ Bredia fordii var. micrantha (C.Chen) R.Zhou & Ying Liu, PhytoKeys 127: 144. 2019.

#### Diagnosis.

Similar to *B. fordii* and *B. esquirolii* in posture and leaf shape, but differs in the stems sparsely pubescent with 1.5–2 mm long, spreading, multiseriate hairs (vs. the former with dense, 2–4 mm long hairs, and the latter with 0.3–1 mm long hairs), young inflorescence bending downwards (vs. erect), slightly curved anthers (vs. geniculate), and cream connectives (vs. yellow at least in the inner stamens). Resembles *B. jiuwanshanensis* in the stems with dense, spreading, 0.3 mm long uniseriate hairs, bending young inflorescence, and isomorphic stamens, but differs markedly in posture (few branched vs. multi-branched), larger (3.5–13 × 1.7–6.3 cm vs. 1.5–7 × 0.7–3.8 cm), submembranous to thin papery (vs. thick papery), more or less ovate leaf blade (vs. elliptic to narrowly elliptic) with cordate base (vs. obtuse or rounded), and acuminate apex (vs. acute), and anthers and connectives of cream color (vs. purple anthers and yellow connectives at anther base).

#### Description.

Shrubs, to 60 cm tall. Stems erect or ascending, few-branched, terete; branchlets densely pubescent with 0.3 mm long uniseriate hairs and sparsely pubescent with 1.5–2 mm long, spreading, multiseriate hairs. Leaves opposite, equal or unequal; petiole 1.8–6 cm long, densely pubescent with spreading, multiseriate and uniseriate hairs; leaf blade ovate-cordate to ovate-lanceolate, rarely obovate, 3.5–13 × 1.7–6.3 cm, submembranous to thin papery, secondary veins 3 on each side of midvein, adaxial surface green, pubescent with bent uniseriate hairs, denser along the veins, abaxial surface pale green or ± purplish, pubescent with spreading, uniseriate and multiseriate hairs, base shallowly cordate, margin ciliate and inconspicuously serrulate with each tooth having a terminal seta, apex acuminate, rarely acute. Inflorescence bending downwards when young, 2–10-flowered; peduncle 1.3–3.3 cm long, densely pubescent. Flowers bisexual, radial but androecium slightly bilateral, 4-merous, pedicles, hypanthium and calyx lobes densely pubescent with 0.5–1 mm long hairs; pedicel 0.8–1.5 cm long; hypanthium yellowish-green, funnel-shaped, 5 × 3–4 mm; calyx lobes 4, linear, 4–5 × 0.5 mm; petals 4, purplish-red, ovate, ca. 8 × 7 mm, puberulent on the abaxial surface with uniseriate hairs, apex oblique; stamens 8 in two whorls, isomorphic, subequal in length with the outer whorl slightly longer than the inner one, filaments ca. 6–7 mm long, bent with the anthers to one side of the flower, anthers lanceolate, slightly curved, 6–7 mm long, cream, connective forming 2 ventral lobes and a dorsal tubercle of the same color; ovary ca. 4 mm long, 2/3 as long as the hypanthium (crown excluded), half inferior, locules 4, apex with membranous crown, crown margin ciliate with dark red glandular hairs; style ca. 1.3 cm long, basally puberulent. Capsule ca. 6 × 5 mm, funnel-shaped, with enlarged apical crown; placentation axial, placentas non-thready. Seeds numerous, cuneate.

#### Phenology.

Flowering July to August, fruiting August to September.

#### Distribution.

*Bredia micrantha* is currently only known from Dushan County, Guizhou, China, occurring among rocks near stream or on moist rock in forests.

#### Additional specimen examined.

**China. Guizhou Province**: • Dushan County, Cha-he to Li-zi-chong, on shaded and moist rocks or rock cliff in forests, 1,000 m, 16 Aug 2019, J.H.Dai and Y.Liu 745 (A, PE, SYS).

### ﻿ Bredia pingshanensis

Taxon classificationPlantaeMyrtalesMelastomataceae

J.H.Dai & Ying Liu sp. nov.

35F50B87-3B27-52CF-A754-2F38CD4666C5

urn:lsid:ipni.org:names:77371616-1

Figs 13, 14, 15D

#### Type.

China • Sichuan: Pingshan County, Lao-jun-shan Nature Reserve, Xin-tian-zui to Er-nian-ping, on shaded and moist steep slope along the road, 1,263 m, 30 Aug 2019, *J.H.Dai and Y.Liu 757* [holotype: PE!; isotypes: A!, SYS!].

#### Diagnosis.

Somewhat resembles *B. cordata* in ovate leaf blade of similar size, glandular pubescent hypanthium, and ovate-triangular calyx lobes, but differs in the stems and leaves inconspicuously puberulent with bent uniseriate hairs (vs. usually with dense spreading hairs), leaves usually unequal (vs. equal to subequal), leaf base very shallowly cordate or rounded (vs. cordate), white (vs. pink) petals with the abaxial surface reddish glandular pubescent along the midvein (vs. glabrous to inconspicuously puberulent, colorless), deep purple anthers (vs. white to light purple), and purplish-red connectives (vs. yellow in the inner stamens).

#### Description.

Shrublets to 35 cm tall. Stems erect or ascending, few-branched, terete; branchlets with bending uniseriate hairs and very sparse ca. 0.3 mm long multiseriate setas. Leaves opposite, often unequal; petiole 0.8–8.2 cm long, indumentum same as branchlets but with denser seta; leaf blade ovate or ovate-elliptic, larger ones 4.5–12.3 × 2.8–7.5 cm, smaller ones 2.2–7 × 1.2–5 cm, membranous, secondary veins 2 or 3 on each side of midvein, adaxial surface green, with bent uniseriate hairs and very sparse seta, abaxial surface pale green, pubescent with bent uniseriate hairs, densely so along veins, base shallowly cordate to rounded, sometimes oblique, margin serrulate with each tooth having a terminal seta, apex short acuminate. Inflorescence terminal, cymose, or cymose paniculate, 3–12-flowered; peduncle 0.8–2.8 cm long, puberulent as branchlets. Flowers bisexual, radial but androecium bilateral, 4-merous, pedicles, hypanthium and calyx lobes densely pubescent with bent uniseriate hairs and 0.5–1 mm long multiseriate glandular hairs; pedicel 0.4–1 cm long; hypanthium purplish-red, funnel-shaped, 5 × 3–4 mm; calyx lobes 4, ovate-triangular, 3–2 × 1 mm; petals 4, white with pink apex, ovate, ca. 7 × 5.5 mm, puberulent along midvein on the abaxial surface with red glandular hairs, apex slightly oblique; stamens 8 in two whorls, dimorphic, unequal in length, with the outer whorl much longer than the inner one, longer stamens ca. 16 mm long, filaments ca. 9 mm long, anthers lanceolate, curved, ca. 7 mm long, purple, connectives decurrent, purplish-red, forming 2 ventral lobes, shorter stamens ca. 9 mm long, filaments ca. 5 mm long, anthers lanceolate, slightly curved, ca. 4 mm, deep purple, connectives purplish-red, forming 2 ventral lobes and a dorsal tubercle; ovary ca. 4 mm long, 2/3 as long as the hypanthium (crown excluded), half inferior, locules 4, apex with membranous crown, crown margin ciliate with red glandular hairs; style ca. 1.2 cm long, basally inconspicuously puberulous. Capsule ca. 6 × 5 mm, funnel-shaped, with enlarged apical crown; placentation axial, placentas non-thready. Seeds numerous, cuneate.

#### Phenology.

Flowering August, fruiting September to October.

#### Etymology.

The specific epithet refers to the type locality of the new species, Pingshan County.

#### Distribution.

*Bredia pingshanensis* is only known from Lao-jun-shan Nature Reserve in Pingshan County, Sichuan, China, occurring on shaded and moist steep slopes along the road at forest margin.

### ﻿Key to species of *Bredia*

**Table:** 

1	Leaf blade 3-veined	**2**
–	Leaf blade 5–9-veined	**3**
2	Creeping shrubs; leaf blade 1.8–3(–4) × 0.5–1.2 cm; stamens isomorphic	***B. guidongensis***
–	Erect shrubs; leaf blade 5–11 × 1.5–2.5(–3.5) cm; stamen dimorphic	***B. oldhamii***
3	Most parts of the stem prostrate, usually less than 20 cm tall	**4**
–	Erect or only lower parts of the stem prostrate, usually above 25 cm tall	**10**
4	Leaves unequal; adaxial leaf surface hispid with spreading stout bristles 2–4 mm long	***B. hispida***
–	Leaves equal or subequal; adaxial leaf surface pubescent, puberulent, rarely hispid with 1 mm long bristles	**5**
5	Stem with appressed hairs	***B. microphylla***
–	Stem with spreading hairs	**6**
6	Leaf blade membranous and fragile; petal margin undulate	***B. enchengensis***
–	Leaf blade papery to stiff papery; petal margin entire	**7**
7	Leaf blade elliptic, to narrowly elliptic, base rounded	***B. jiuwanshanensis***
–	Leaf blade ovate to orbicular, base cordate	**8**
8	Stamens dimorphic	***B. rotundifolia***
–	Stamens isomorphic	**9**
9	Leaves distichous opposite in the upper part of stem; inflorescence 1–2-flowered	***B. changii***
–	Leaves decussate in the upper part of stem; inflorescence 1–8-flowered	***B. repens***
10	Leaf veins adaxially strongly sunken with interveinal areas bullate each with an apical seta	***B. bullata***
–	Leaf veins and interveinal areas not as described above	**11**
11	Petal margin undulate	**12**
–	Petal margin entire	**17**
12	Abaxial leaf surface with minute yellowish glandular hairs	**B. esquirolii var. esquirolii**
–	Abaxial leaf surface without minute yellowish glandular hairs	**13**
13	Stems densely pubescent with reddish multiseriate hairs	**B. esquirolii var. rubra**
–	Stems glabrescent, sparsely villous, or densely pubescent with uniseriate hairs	**14**
14	Hypanthium setose, hair multiseriate	**B. macrophylla var. macrophylla**
–	Hypanthium puberulous with uniseriate hairs	**15**
15	Stem 4-sided and sulcate	**B. macrophylla var. pulchella**
–	Stem terete	**16**
16	Stem and leaves densely pubescent; calyx lobes triangular to semiorbicular; petals purplish-red	***B. malipoensis***
–	Stem and leaves glabrescent when mature; calyx lobes ovate-elliptic or elliptic; petals white	***B. nitida***
17	Calyx lobes reniform	***B. reniformis***
–	Calyx lobes triangular to linear or less than 1 mm	**18**
18	Stamen dimorphic	**19**
–	Stamen isomorphic	**24**
19	Leaf base cuneate, rarely obtuse	***B. gibba***
–	Leaf base cordate, rarely rounded	**20**
20	Stems inconspicuously puberulent with bending uniseriate hairs; anther connectives are of the same color (purple) in both whorls	***B. pingshanensis***
–	Stems usually pubescent with spreading hairs; outer and inner anther connectives of different color (light purple vs. yellow)	**21**
21	Calyx lobes triangular; inner anthers yellow	**22**
–	Calyx lobes narrowly triangular to lanceolate; inner anthers light purple	**23**
22	Petals pink, upper leaf surface densely pubescent	**B. hirsuta var. hirsuta**
–	Petals white, upper leaf surface glabrous or sparsely setose	**B. hirsuta var. scandens**
23	Stem densely pubescent with 0.5–1 mm long hairs	***B. cordata***
–	Stem villous with 3 mm long hairs	***B. tuberculata***
24	Stem and leaves only pubescent with bent uniseriate hairs	**25**
–	Stem and leaves more or less pubescent with spreading hairs	**26**
25	Calyx lobes less than 1 mm	***B. gracilis***
–	Calyx lobes lanceolate, ca. 5 mm long	***B. plagiopetala***
26	Inflorescence a panicle composed of monochasium	***B. violacea***
–	Inflorescence umbellate or cymose panicle composed of dichasium	**27**
27	Young inflorescences bending downwards	***B. micrantha***
–	Young inflorescences erect	**28**
28	Lower parts of stem prostrate with adventitious roots	**29**
–	Stem erect	**31**
29	Anthers geniculate	***B. fordii***
–	Anthers slightly curved	**30**
30	Stems and leaves with sparse multiseriate hairs	***B. longiloba***
–	Stems and leaves with dense multiseriate hairs	***B. yunnanensis***
31	Stem with 3–5 mm long spreading hairs; anthers geniculate	***B. fordii***
–	Stem with ca. 0.5 mm long spreading hairs; anthers slightly curved	**32**
32	Mature leaves ± glabrescent; all main veins basal	***B. cordata***
–	Mature leaves pubescent; the inner most pair of veins often diverged above the base	***B. velutina***

## Supplementary Material

XML Treatment for Bredia bullata

XML Treatment for Bredia cordata

XML Treatment for Bredia esquirolii

XML Treatment for Bredia esquirolii var. rubra

XML Treatment for Bredia fordii

XML Treatment for Bredia hirsuta

XML Treatment for Bredia jiuwanshanensis

XML Treatment for Bredia macrophylla

XML Treatment for Bredia macrophylla var. pulchella

XML Treatment for Bredia micrantha

XML Treatment for Bredia pingshanensis

## ﻿Additional information

### Use of AI

No use of AI was reported.

### Author ORCIDs

Jin-Hong Dai https://orcid.org/0000-0001-5069-6016

Zhe Zhong https://orcid.org/0009-0001-8967-6036

Ren-Chao Zhou https://orcid.org/0000-0002-5836-8583

Ying Liu https://orcid.org/0000-0003-0613-837X

### Data availability

All of the data that support the findings of this study are available in the main text or Supplementary Information.

## Supplementary materials

Supplementary material 1 Source of materials studied Data type xlsx Explanation note The voucher of accessions with possible hybrid origin is followed by asterisks. https://binary.pensoft.net/file/1459079This dataset is made available under the Open Database License (http://opendatacommons.org/licenses/odbl/1.0/). The Open Database License (ODbL) is a license agreement intended to allow users to freely share, modify, and use this Dataset while maintaining this same freedom for others, provided that the original source and author(s) are credited.Jin-Hong Dai, Zhe Zhong, Ren-Chao Zhou, Ying Liu
